# Malaria cases in China acquired through international travel, 2013–2022

**DOI:** 10.1093/jtm/taae056

**Published:** 2024-04-09

**Authors:** Yan Zhu, Angela Cadavid Restrepo, Hai-Bo Wang, Deborah J Mills, Rong-Rong Liang, Zhi-Bin Liu, Colleen L Lau, Luis Furuya-Kanamori

**Affiliations:** UQ Centre for Clinical Research, Faculty of Medicine, The University of Queensland, Building 71/918 RBWH Herston, Brisbane, QLD 4029, Australia; Zhuhai International Travel Healthcare Center of China Customs, 133 Qiaoguang Rd, Xiangzhou District, Zhuhai, 519000, China; UQ Centre for Clinical Research, Faculty of Medicine, The University of Queensland, Building 71/918 RBWH Herston, Brisbane, QLD 4029, Australia; Zhuhai International Travel Healthcare Center of China Customs, 133 Qiaoguang Rd, Xiangzhou District, Zhuhai, 519000, China; Dr Deb The Travel Doctor, Travel Medicine Alliance, 5/247 Adelaide St, Brisbane, QLD 4000, Australia; Guangzhou International Travel Healthcare Center of China Customs, 207 Longkou West Rd, Tianhe District, GuangZhou, 510635, China; Fangshan District Center for Disease Control and Prevention, 1 Baiyang East Rd, Fangshan District, Beijing, 102401, China; UQ Centre for Clinical Research, Faculty of Medicine, The University of Queensland, Building 71/918 RBWH Herston, Brisbane, QLD 4029, Australia; UQ Centre for Clinical Research, Faculty of Medicine, The University of Queensland, Building 71/918 RBWH Herston, Brisbane, QLD 4029, Australia

**Keywords:** Malaria, disease surveillance, travel medicine

## Abstract

**Background:**

Despite the World Health Organization certifying China malaria-free in 2021, the risk of local transmission caused by imported malaria cases remains a significant clinical and public health issue. It is necessary to present the changing trends of malaria in China and discuss the role of travel medicine services in consolidating malaria elimination.

**Methods:**

This study systematically reviewed articles and reports related to human malaria from 2013 to 2022 published in international and Chinese databases. Data on malaria (i.e. number of cases, *Plasmodium* spp., diagnostic method, country of acquisition, provinces with high risk of re-introduction and transmission) were collected and synthesized, then summarized using descriptive statistics.

**Results:**

Overall, 24 758 cases of malaria (>99.5% laboratory confirmed, > 99.2% imported, 0.5% fatal) were reported in China from 2013 to 2022, with a downward trend over the years (4128 cases in 2013 compared to 843 cases in 2022; *χ*^2^ trend *P* = 0.005). The last locally acquired case was reported in 2017. *Plasmodium falciparum* (65.5%) was the most common species identified, followed by *P. vivax* (20.9%) and *P. ovale* (10.0%). Two *P. knowlesi* cases were also identified in 2014 and 2017 in returned travellers from Malaysia and Indonesia, respectively. The most common countries for malaria acquisition were Ghana, Angola and Myanmar. *Plasmodium vivax* was mainly detected in returned travellers from Myanmar, while *P. falciparum* and *P. ovale* were detected in travellers from sub-Saharan Africa. Imported cases were mainly reported in Yunnan, Jiangsu, Sichuan, Guangxi, Shandong, Zhejiang and Henan provinces, where large numbers of Chinese people travel overseas for work.

**Conclusion:**

Returned travellers from malaria-endemic countries pose a significant risk of malaria re-introduction to China. Travel medicine should be strengthened to improve the capacity and accessibility of both pre- and post-travel services, including malaria prophylaxis and prompt diagnosis of illness in returned travellers.

## Introduction

Malaria has been one of the most serious and fatal infectious diseases in human history. In 2021, nearly half of the world’s population was at risk of malaria with an estimation of 247 million cases and 619 000 deaths worldwide.^1^

In China, malaria is a notifiable disease.^2^ Detailed information on malaria cases is required to be reported to China CDC in a standard format (containing name, national identification number, age, sex, residential address, occupation, symptoms, type of case diagnosis [suspected/clinically diagnosed/confirmed cases], diagnosis, type of species, date of onset, time of diagnosis, travel history) through China Information System for Disease Control and Prevention^2^ by the medical institution (i.e. hospitals and clinics) who initially confirms the diagnosis.^3^ Then, the case will be followed up by the local municipal CDC to conduct further investigation and confirmation in accordance with China’s National Malaria Elimination Work Plan.^4^ At present, national-level operations (i.e. policy development and data management) are operated by China CDC (Beijing), while provincial and municipal CDCs are responsible for their region. China’s approach requires case reporting within 1 day of case detection, case investigation and classification within 3 days and targeted and tailored response activities within 7 days.^5^ It is supported by a real-time, web-based malaria elimination reporting platform developed in 2003 for the severe acute respiratory syndrome based on China Information System for Disease Control and Prevention (accessed by authorised personnel only via virtual private network).^6^

China in the past had a high incidence of malaria, which not only posed a serious health hazard but also placed a substantial burden on the national medical resources. However, through an ongoing commitment over 70 years, China transitioned from reporting 30 million malaria cases per year in the 1940s to achieving a ‘zero indigenous cases’ milestone in 2017.^7^ On June 30, 2021, China received the Malaria Elimination Certification from the World Health Organisation (WHO), joining the rank as the 40th nation globally.^8^ This accolade is etched as a landmark achievement in China’s public health history.

Despite successfully achieving malaria elimination, China still faces the continuous risk of imported cases by international travellers.^7^^,^^9^^,^^10^ The continuous growth in international travel (98.18 million Chinese citizens travelled abroad and 26.29 million foreigners travelled to China in 2013, growing to 169.2 million and 49.11 million, respectively, in 2019^11^) significantly raises the risk of importation and re-introduction of malaria. Travel medicine is an emerging medical field in China. Travel medicine services are provided by only 42 International Travel Healthcare Centers (ITHCs) of the General Administration of Customs of the People’s Republic of China (China Customs),^12^, and there is a limited number of travel medicine clinics operated by infectious disease departments in tertiary hospitals (e.g. Huashan Hospital of Fudan University, Shanghai Public Health Clinical Centre, Jiangsu Provincial People’s Hospital and Beijing Youan Hospital).^13^ Therefore, the capacity of travel medicine services in China is relatively scarce compared to the travel volume and the size of the country.

This study aims to describe the epidemiological trends in malaria over the past decade in China, investigate the potential risk of malaria importation and recurrence of local transmission and discuss the need for improving access and provision of travel medicine services and advice for most travellers.

## Methods

### Data sources and eligibility criteria

No primary data was used. Aggregated data were extracted from published articles for this study.

We conducted a systematic search of published articles on human malaria in China from 2013 to 2022 in academic databases and Chinese-specific databases, as well as grey literature. Five databases (China National Knowledge Infrastructure [CNKI],^14^ Wanfang Data,^15^ Chinese Medical Journal Network,^16^ Google Scholar, PubMed) were searched using the terms ‘malaria’ and ‘China’ as keywords (search strategies are provided in Supplementary material S1). Grey literature included government reports from the China National Health Commission (www.nhc.gov.cn), China National Disease Control and Prevention Administration (www.ndcpa.gov.cn), China CDC (www.chinacdc.cn) and China Customs (www.customs.gov.cn). Links to the government reports are provided in Supplementary material S2.

Articles were included for data extraction and synthesis if all of the inclusion criteria were met: (i) peer-reviewed articles or official Chinese government reports; (ii) malaria data were collected from the China Information System for Disease Control and Prevention and (iii) articles or reports presented malaria data on a national level or specific risk groups that were represented in China.

Articles were excluded if: (i) they were case reports, case series, editorials, commentaries or policy documents; (ii) articles or reports with malaria data relating only to specific provinces and/or cities, with the exception of the ones identifying the specific risk groups of travellers.

Two researchers (Y.Z., Z.B.L.) performed the literature search and independently screened the articles and extracted the data. When potential duplicate records were identified by either of the two reviewers or any discrepancies arose, these were decided after discussion with a third researcher (L.F.K.). After deleting duplicates, titles and abstracts were checked for eligibility for inclusion. After the first eligibility check, the full text was screened.

### Data extraction and synthesis

Data on cases (i.e. clinically diagnosed cases, laboratory-confirmed cases, severe cases and deaths), type of cases (i.e. locally acquired or imported), etiological agent (*Plasmodium* species), country of acquisition and province with risk of re-introduction (i.e. reported cases, malaria elimination status) were extracted and recorded on Excel spreadsheet.

Due to the included articles only provided cases from the top five countries of acquisition and provinces per year (representing the range from 51.8 to 91.2% and from 41.2 to 63.4% of the total cases separately), we summarized the data by calculating cumulative rankings in countries of acquisition and provinces. Mixed infections of *Plasmodium* species and cases of special malaria cases were categorized separately for statistical analyses. In addition, we assessed the risk of malaria re-introduction by considering malaria transmission (i.e. reported cases, species and density of mosquitoes) and public health capacity (i.e. achievements in local malaria elimination).

### Statistical analysis

The extracted data were summarized using descriptive analyses and presented in tables, figures and maps. We evaluated the trend in number of cases and deaths over the years using the chi-squared (χ^2^) test for trend. Statistical analyses were conducted in Stata MP 17 (StataCorp, TX). ArcGIS software 10.5.1 was used to create maps (ESRI, Redlands, CA).

## Results

Overall, 558 articles and 16 government reports were retrieved, of which 19 records (all published in peer-reviewed journals) met the inclusion criteria and were included in the analysis.^9^^,^^10^^,^^17–33^ None of the resources from the grey literature search (i.e. government reports) were included, as they did not report stratified data by country of acquisition.

Of the 19 included publications, 17 were published in peer-reviewed journals by researchers/staff from China CDC and written in Chinese.^17–33^ Thirteen of these 17 records were published in *Chinese Journal of Parasitology and Parasitic Diseases* (journal sponsored by the Chinese Society of Preventive Medicine and China CDC),^17–26^^,^^28^^,^^30^^,^^33^ two were published in *China Tropical Medicine,*^29^^,^^32^ one in *Journal of Tropical Diseases and Parasitology*^27^ and one in *Journal of Pathogen Biology.*^31^

Ten of these 17 publications reported annual surveillance data in China^17–26^ (number of cases, incidence by provinces, epidemiological characteristics of the cases [e.g. imported vs local acquired, severity of the cases], *Plasmodium* spp). The remaining seven publications were epidemiological papers/publications: (i) assessing risk factors for malaria infection in returned Chinese travellers^29^^,^^32^; (ii) evaluating the effectiveness of surveillance response system^27^^,^^28^; (iii) identifying high risk travel groups^30^^,^^31^ and (iv) quantifying the risk of re-introduction and further transmission in the provinces.^33^ Two articles by Fang *et al.*^10^ and Wu *et al.*^9^ were published in English and contributed evidence on the characteristics of high-risk groups of travellers and countries of acquisition.

### Total malaria cases and trend

From 2013 to 2022, 24 758 cases of malaria (>99.5% laboratory confirmed) and 131 malaria-related deaths (0.5% fatality rate) were reported in China. Over 93% of the cases were reported by Chinese citizens. From 2020 onwards, all cases were laboratory-confirmed by microscopy, rapid diagnostic test (RDT) or polymerase chain reaction (PCR). No more cases were diagnosed by clinical symptoms plus epidemiological history.

**Figure 1:**
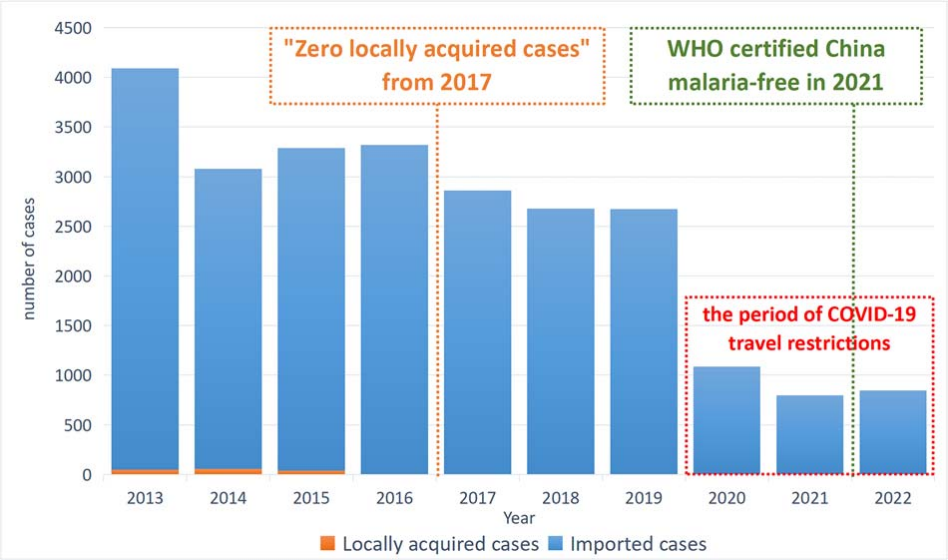
Trend in locally acquired and imported malaria cases in China from 2013 to 2022. Note: the number of locally acquired cases from 2013 to 2016 was 48, 56, 40 and 3, respectively

A significant downward trend in the number of cases (χ^2^ trend *P* = 0.005) and deaths (χ^2^ trend *P* = 0.007) was observed over the years (Table 1; Figure 1). Over 99.2% of cases were imported, and from 2017, no locally acquired cases were reported (Figure 1). Prior to COVID-19 (2013–2019), a downward trend was observed in imported cases (*χ*^2^ trend *P* = 0.044) and in *P. vivax* cases (*χ*^2^ trend *P* = 0.018). The trend for *P. falciparum* (*χ*^2^ trend *P* = 0.162) and *P. ovale* (*χ*^2^ trend *P* = 0.054) was not statistically significant, while *P. malariae* showed an upward trend (*χ*^2^ trend *P* = 0.029) (Supplementary material S3). No statistically significant trends were observed from 2020 to 2022.

**Table 1:** Total number of malaria cases by type of diagnosis, and severe cases and death in China from 2013 to 2022

Year	Total	Laboratory-confirmed cases *n* (%)	Severe cases *n* (%)	Deaths *n* (%)
2013	4128	4087 (99)	156 (3.8)	23 (0.6)
2014	3078	3057 (99.3)	170 (5.5)	25 (0.8)
2015	3288	3265 (99.3)	163 (5)	20 (0.6)
2016	3321	3306 (99.5)	185 (5.6)	15 (0.5)
2017	2861	2852 (99.7)	136 (4.8)	7 (0.2)
2018	2678	2673 (99.8)	117 (4.4)	7 (0.3)
2019	2674	2669 (99.8)	*n/a*	19 (0.7)
2020	1086	1086 (100)	*n/a*	6 (0.6)
2021	799	799 (100)	*n/a*	3 (0.4)
2022	845	845 (100)	36 (4.3)	6 (0.7)
Total	24 758	24 639 (99.5)	/	131 (0.5)

*n/a* data were unavailable.

*Laboratory-confirmed:* microscopy, RDT or PCR.^38^

*Severe case:* coma, severe anaemia, acute renal failure, pulmonary oedema, acute respiratory distress syndrome, circulatory failure or shock and/or metabolic acidosis.^39^

### *Plasmodium* species in imported cases

**Figure 2:**
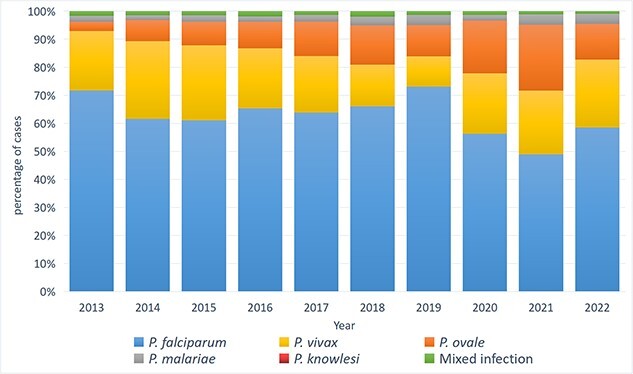
Proportion of *Plasmodium* species of imported malaria cases in China from 2013 to 2022

*Plasmodium falciparum* was the predominant species accounting for 65.5% of cases, followed by *P. vivax* (20.9%) and *P. ovale* (10.0%). The annual number of *P. falciparum* (from 2899 in 2013 to 494 in 2022) and *P. vivax* (from 859 in 2013 to 204 in 2022) cases declined over time, while the number of cases of the other species remained stable (Table 2; Figure 2, Supplementary material S3). Two cases of *P. knowlesi* were identified in Guangdong province: one in 2014 acquired from Malaysia and the other in 2017 from Indonesia.

**Table 2:** Distribution of *Plasmodium* spp. of imported malaria cases in China form 2013 to 2022 (does not include locally transmitted cases)

Year	Imported cases	*Plasmodium* spp.						
		*P. falciparum*	*P. ovale*	*P. malariae*	*P. vivax*	*P. knowlesi*	Mixed	Clinically diagnosed cases
2013	4042	2899	133	51	859	0	65	35
2014	3021	1876	231	52	798	1	44	19
2015	3248	1991	272	70	851	0	47	17
2016	3317	2157	311	64	709	0	61	15
2017	2858	1819	352	67	573	1	37	9
2018	2671	1763	376	82	393	0	52	5
2019	2673	1950	298	96	289	0	35	5
2020	1085	610	204	22	234	0	15	0
2021	798	390	187	30	182	0	9	0
2022	844	494	108	30	204	0	8	0

*Mixed:* more than one *Plasmodium* spp. was detected.

*Clinically diagnosed cases:* history of travelling to malaria-endemic area during the malaria transmission season or history of blood transfusion within the last 2 weeks, with typical clinical symptoms of malaria but negative microscopy and RDT results.^38^

There were six cases of transfusion-related malaria, all caused by *P. falciparum.* In addition, there were four introduced *P. vivax* cases without local transmission in 2018; and five cases of long-term dormant *P. malariae* in Guangdong province (Supplementary material S4).

### Main countries of acquisition

**Figure 3:**
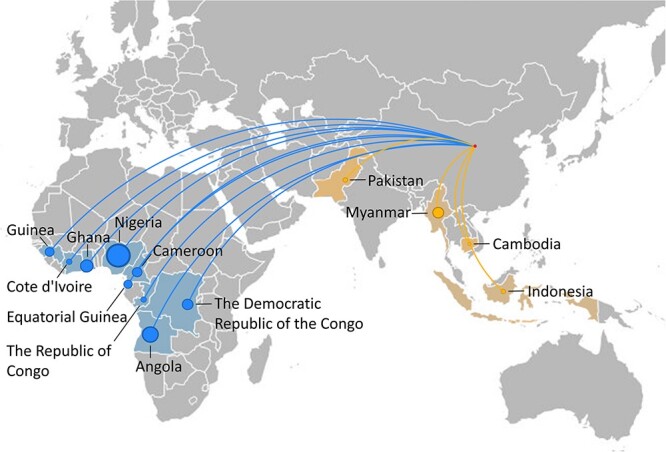
Main countries of acquisition of imported malaria cases from Africa (blue, mainly *P. falciparum*) and Asia (yellow, mainly *P. vivax*) into China from 2013 to 2022. The size of the circle is proportional to the number of imported cases into China. Map is for illustrative purposes only and not for territory claims

Malaria cases caused by *P. falciparum* and *P. ovale* were mainly acquired in central and west African countries (e.g. Nigeria, Angola, Ghana, Guinea, Cameroon), whereas cases of *P. vivax* were mainly acquired in Asia (e.g. Myanmar, Indonesia and Pakistan). The majority of cases were diagnosed in Chinese labourers working in Nigeria, Angola, Ghana and Myanmar (Figure 3, Supplementary material S5).

### Provinces with higher risk of re-introduction and further transmission

**Figure 4:**
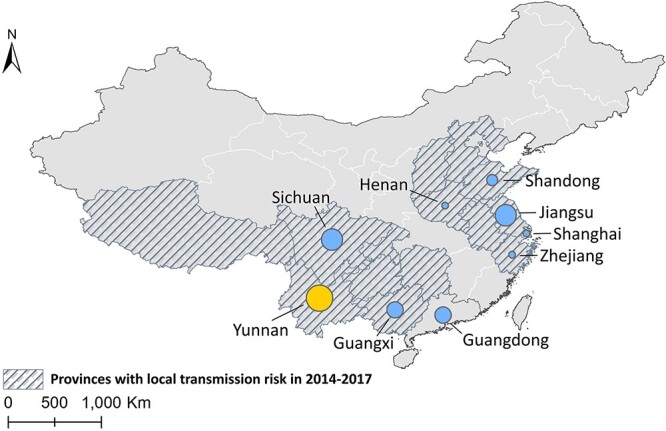
Provinces with the highest number of malaria cases reported from 2013 to 2022 The size of the circle is proportional to the number of imported cases. Yellow and blue dots indicate cases mainly imported from South-East Asia and Africa, respectively. Shaded areas indicate provinces with predicted transmission risk from 2014 to 2017 based on incidence of malaria, density of vectors and implementation of malaria elimination strategy 21-23,33. Map is for illustrative purposes only and not for territory claims

Imported cases were mainly reported in Yunnan, Guangxi, Jiangsu, Sichuan, Shandong, Zhejiang and Henan provinces. All these provinces were reported to have been locally acquired until 2017 and therefore possess competent vector species and suitable environmental conditions for malaria transmission (Figure 4, Supplementary material S6).

## Discussion

In this study, we found that in the last decade, malaria cases in China have been predominantly imported (99.2%). There was an overall moderate decrease trend in the number of cases, with a moderate decrease from 2013 to 2019 and a sharp decline from 2020 to 2022. The sharp decline was likely to be largely attributable to the severe travel restrictions imposed in response to the COVID-19 pandemic. But there was no downward trend in the numbers of *P. falciparum* and *P. ovale* before COVID-19, suggesting the risk of imported cases from Africa into China. More accurate reflection of the current risk of importation would require a review of data from 2023 onwards, when cases are expected to increase due to opening of international borders.

The vast majority of imported cases were among Chinese citizens returning from Nigeria, Angola, Ghana, Guinea and Cameroon (for *P. falciparum* and *P. ovale* cases) and Myanmar (for *P. vivax* cases), which reflects China’s labour export programmes. For example, Longhui County (Hunan province) had high incidence of imported *P. vivax*,^34^ and these cases have been epidemiologically linked to gold miners from this county working in Myanmar and Indonesia. Likewise, in 2013, Shanglin County (Guangxi province) reported 1052 *P. falciparum* cases in Chinese gold miners returning from Ghana, accounting for 25.5% of the total malaria cases in China in that year.^19^ More recently, in 2022, four cities across three provinces (i.e. Beijing, Shenzhen [Guangdong province], Kunming [Yunnan province] and Hangzhou [Zhejiang province]) reported 95 *P. falciparum* cases in total, in Chinese railway, waterway and port construction workers returning from Guinea.^29^ With the implementation of China’s ‘The Belt and Road’ policy, more Chinese workers will participate in infrastructure projects and business investment in Africa and South East Asia, which may increase the risk of malaria importation.

Yunnan, which shares a border with Myanmar, is the province facing the highest risk of the *P. vivax* re-transmission in China, not only because it had the highest incidence and number of cases of imported *P. vivax* in China resulting from the close business connections with Myanmar^17–27^^,^^32^ but also the potential risk of residents in China being infected by malaria-carrying mosquitoes from Myanmar considering the absence of natural barriers in the China–Myanmar border region.^27^^,^^28^^,^^32^ Other major labour exporting provinces include Sichuan, Jiangsu, Guangxi, Shandong, Zhejiang and Henan which are also at high risk of re-introduction because of the presence of imported cases as well as the local environmental suitability for transmission. The risk of local transmission was relatively low in Guangdong Province and Shanghai Municipality, where most reported cases were reported by transit travellers, given that these two regions were important air-entry points into China and the quarantine isolation policy during COVID-19.^17^^,^^18^^,^^25^

To address the risk of malaria re-introduction by returned travellers from different malaria-endemic regions, the implementation of travel medicine services would provide obvious benefits. There are two separate travel medicine services in China (i.e. travel consultation and health certificate). For now, the large majority of pre-travel consultations in China are carried out by the International Travel Healthcare Centers of China Customs (ITHCs) because the health certificate ‘Certificate of Health Examination for International Travellers’ (containing medical examination and laboratory tests against infectious diseases) is compulsory for Chinese citizens planning to travel abroad for more than one year. Travellers can opt for free of charge travel consultations at the ITHCs which include education and awareness of disease prevention measures and advice on vaccines and chemoprophylaxis. Furthermore, pre-travel consultations by ITHC are available and free of charge for all travellers regardless of the trip duration. The pre-travel consultations and pre-departure health examination services cover the vast majority of malaria high-risk groups and make it possible for the country to setup a health promotion framework that helps international travellers recognising the risk of infectious diseases at destinations, provide preventive measures (e.g. personalised risk assessment, vaccine recommendations and malaria chemoprophylaxis), and schedule health education and medical follow-up at the national level.

However, there are still some limitations with travel medicine services in China. Lin *et al*.^30^ and Zhang *et al.*^35^ examined the use of pre- and post-travel consultation and malaria chemoprophylaxis among Chinese travellers to and returning from high risk destinations for malaria, and found deficiencies in both awareness and protective behaviours. Only 4% of travellers received information about malaria from the travel health providers,^35^ and alarmingly, less than 5% had taken malaria chemoprophylaxis.^30^^,^^35^ These gaps may be attributable to many factors including policy constraints and economic considerations. For instance, some popular antimalarial chemoprophylaxis medications (e.g. Malarone) have not yet been granted pharmaceutical registration approval in China. The ‘Guidelines for malaria diagnosis and treatment’^36^ published by the Expert Group of the National Center for Infectious Diseases of China in 2022 explicitly recommends only one drug (i.e. piperaquine phosphate) for malaria chemoprophylaxis and advised against its continuous use for more than three months. In addition, the provision of travel medicine services and the development of expertise in this field have yet to receive adequate attention from the general public, clinicians and the government. Despite free travel consultation service being available in ITHCs, only a minority of travellers accessed the service, and both pre- and post-travel consultations are not sufficiently publicised to the travelling public. Moreover, travel vaccinations and malaria chemoprophylaxis, and even pre-travel consultations in hospitals have not been integrated into public healthcare expenditure, so travellers have to bear these expenses (with the exception of the Yellow Fever vaccine).

For a complete risk assessment, detailed line-listed information about the cases (e.g. demographic information, specific case number by country of acquisition, use of malaria chemoprophylaxis, length of staying abroad) is needed; however, this is not publicly available. Nonetheless, we provide valuable information about the top high-risk countries and present a clear picture of the epidemiological state of malaria from a travel medicine perspective. In the annual surveillance publications included in the current study,^17–26^ only cases from the top five countries of acquisition and provinces per year were reported (presenting the 63.8 and 51.5% of the total cases separately). Although our findings were consistent with previous research,^9^^,^^10^^,^^27^^,^^28^ there were countries and provinces with small number of cases that were not included in the reports and therefore omitting from our assessment of important risk and re-introduction of transmission. In the data collected, mixed infections were considered as a separate category for statistical purposes, and even if a case of mixed infection included specific species (i.e. mix of *P. falciparum* and *P. ovale*), the case was only counted as one mixed infection. However, because of the small number of mixed infection cases, the impact on the overall risk assessment was likely limited.

Although China has achieved the goal of malaria elimination, thousands of imported malaria cases each poses a risk of re-introduction of malaria. In response to this risk, travel medicine services and surveillance systems should align with the technical requirements of the WHO Malaria Program (i.e. prevention, treatment, surveillance, elimination, and reporting).^37^ Strengthening the capacity of China’s travel medicine services and travellers’ awareness of the services will improve the health safety of international travellers and also protect China’s achievements in malaria elimination.

## Supplementary Material

Supplementary_material_taae056

## Notes

Preliminary results of the study were presented at the 6th Southern Cross Travel Medicine Conference and the 2nd Southern Cross Tropical Medicine Conference, Sydney, Australia (Sept 1–3, 2023).

## References

[1] World Health Organization . World Malaria Report 2022. 2022. https://www.who.int/publications/i/item/9789240064898 (6 September 2023, date last accessed).

[2] China National Health Commission . Specification for the Reporting Information on Infectious Disease. 2015. http://www.nhc.gov.cn/jkj/s3577/201511/f5d2ab9a5e104481939981c92cb18a54.shtml (6 September 2023, date last accessed).

[3] Chinese Centre for Disease Control and Prevention . Technical Guidelines for National Infectious Disease Reporting Management. 2016. http://www.kaifu.gov.cn/zfxxgk/zssyhqtdw_18815/qjkzx/zcwj_19513/201911/t20191102_6722566.html (9 September 2023, date last accessed).

[4] China National Health Commission . National Malaria Elimination Action Plan (2010-2020). 2020. https://www.gov.cn/zwgk/2010-05/26/content_1614176.htm (6 September 2023, date last accessed).

[5] Cao J, Sturrock HJ, Cotter C et al. Communicating and monitoring surveillance and response activities for malaria elimination: China's "1-3-7" strategy. PLoS Med 2014; 11:e1001642. 24824170 10.1371/journal.pmed.1001642 PMC4019513

[6] Cao J, Newby G, Cotter C et al. Achieving malaria elimination in China. Lancet Public Health 2021; 6:e871–2. 34838192 10.1016/S2468-2667(21)00201-2 PMC9022785

[7] World Health Organization . From 30 Million Cases to Zero: China is Certified Malaria-Free by WHO. 2021. https://www.who.int/news/item/30-06-2021-from-30-million-cases-to-zero-china-is-certified-malaria-free-by-who (6 September 2023, date last accessed).

[8] The Xinhua News Agency . China has been Certified as Having Eliminated Malaria, Another Milestone in the Country's Health History. 2021. https://www.gov.cn/xinwen/2021-07/10/content_5623932.htm (6 September 2023, date last accessed).

[9] Wu Y, Liu M-Y, Wang J-L et al. Epidemiology of imported infectious diseases, China, 2014–18. J Travel Med 2020; 27:taaa211. 33283238 10.1093/jtm/taaa211 PMC7757385

[10] Fang L-Q, Sun Y, Zhao G-P et al. Travel-related infections in mainland China, 2014–16: an active surveillance study. Lancet Public Health 2018; 3:e385–94. 30033200 10.1016/S2468-2667(18)30127-0 PMC7164813

[11] National Bureau of Statistics of China . Data on Travel. 2023. https://data.stats.gov.cn/easyquery.htm?cn=C01&zb=A0K01&sj=2019 (6 September 2023, date last accessed).

[12] General Administration of Customs of China . List of International Travel Healthcare Centers in China. 2021. http://wss.customs.gov.cn/wss/ggfw22/3602249/index.html (accessed September 6th 2023).

[13] Wang X-Y, Zhang W-H. Connecting with the international community and taking the Chinese path: the present and future of travel medicine in China. Tourism Tribune 2022; 37:3–6.

[14] China National Knowledge Infrastructure . https://chn.oversea.cnki.net/index/ (6 September 2023, date last accessed).

[15] WanFang Data . https://w.wanfangdata.com.cn/index.html?index=true (6 September 2023, date last accessed).

[16] Chinese Medical Journal Network . https://www.medjournals.cn/index.do (6 September 2023, date last accessed).

[17] Zhang L, Yi B-Y, Yin J-H, Xia Z-G. Epidemiological characteristics of malaria in China, 2022. Chin J Parasitol Parasit Dis 2023; 41:137–41.

[18] Zhang L, Yi B-Y, Xia Z-G, Yin J-H. Epidemiological characteristics of malaria in China, 2021. Chin J Parasitol Parasit Dis 2022; 40:135–9.

[19] Zhang L, Feng J, Xia Z-G. Malaria situation in the People’s republic of China in 2013. Chin J Parasitol Parasit Dis 2014; 32:407–13. 25902667

[20] Zhang L, Zhou S-S, Feng J, Fang W, Xia Z-G. Malaria situation in the People’s Republic of China in 2014. Chin J Parasitol Parasit Dis 2015; 33:319–26. 26931033

[21] Zhang L, Feng J, Zhang S-S, Xia Z-G, Zhou S-S. Malaria situation in the People’s Republic of China in 2015. Chin J Parasitol Parasit Dis 2016; 34:477–81. 30141586

[22] Zhang L, Feng J, Zhang S-S, Jiang S, Xai Z-G, Zhou S-S. Malaria situation in the People’s Republic of China in 2016. Chin J Parasitol Parasit Dis 2017; 35:515–9.

[23] Zhang L, Feng J, Zhang S-S, XIa Z-G, Zhou S-S. The progress of national malaria elimination and epidemiological characteristics of malaria in China in 2017. Chin J Parasitol Parasit Dis 2018; 36:201–9.

[24] Zhang L, Feng J, Zhang S-S, Xia Z-G, Zhou S-S. Epidemiological characteristics of malaria and the progress towards its elimination in China in 2018. Chin J Parasitol Parasit Dis 2019; 37:241–7.

[25] Zhang L, Feng J, Tu H, Yin J-H, Xia Z-G. Malaria epidemiology in China in 2020. Chin J Parasitol Parasit Dis 2021; 39:195–9.

[26] Zhang L, Feng J, Xia Z-G, Zhou S-S. Epidemiological characteristics of malaria and progress on its elimination in China in 2019. Chin J Parasitol Parasit Dis 2020; 38:133–8.

[27] Yin J-H, Xia Z-G. Consolidating the achievements of elimination and preventing reestablishment of transmission: Main challenges and priorities of malaria prevention and control in post-elimination era in China. J Trop Dis Parasitol 2022; 20:241–4+99.

[28] Xia Z-G, Feng J, Zhang L et al. Achieving malaria elimination in China: analysis on implementation and effectiveness of the surveillance-response system. Chin J Parasitol Parasit Dis 2021; 39:733–41.

[29] Zhang L, Yin J-H, Xia Z-G. Risks of and response to cluster outbreak of imported malaria during malaria post-elimination phase in China. China Trop Med 2022; 20:585–9.

[30] Lin K-M, Duo-Quan W, Li S-Z et al. Risk factors of malaria infection in people returned from a travel to Africa in Shanglin, Guangxi. Chin J Parasitol Parasit Dis 2019; 37:539–44.

[31] Feng X-Y, Shi W-Q, Li J-L, Chen J-S, Li Z-X, Xia Z-G. An investigation of malaria vectors in Longhui County, Hunan Province. J Pathogen Biol 2020; 15:317–21.

[32] Feng J, Zhang L, Tu H, Zhou S-S, Xia Z-G. From elimination to post-elimination: characteristics, challenges and re-transmission preventing strategy of imported malaria in China. China Trop Med 2021; 21:5–10.

[33] Zhou X-N, Zhang S-S, Xu J-F et al. Risk assessment for malaria elimination in P. R. China. Chin J Parasitol Parasit Dis 2014; 32:414–8. 25902668

[34] Xiao L . Analysis and countermeasures of the malaria epidemic in Longhui County, 2008-2011. Pract Prev Med 2014; 21:202–3.

[35] Zhang M, Liu Z-Y, He H-T et al. Knowledge, attitudes, and practices on malaria prevention among Chinese international travelers. J Travel Med 2011; 18:173–7. 21539656 10.1111/j.1708-8305.2011.00512.x

[36] Diseases EGoNCfI . Guidelines for malaria diagnosis and treatment. Chin J Parasitol Parasit Dis 2022; 40:419–27.

[37] World Health Organization . Global Malaria Programme. 2023. https://www.who.int/teams/global-malaria-programme (6 September 2023, date last accessed).

[38] Chinese Center for Disease Control and Prevention . National Malaria Elimination Monitoring Program of China. 2012. https://www.chinacdc.cn/jkzt/crb/gjfd/zl/nj/jc/201207/t20120710_64144.html?eqid=ddd4ff640006aea700000002648abfe5 (6 September 2023, date last accessed).

[39] China National Health Commission . Diagnosis of malaria (WS 259-2015). 2015. http://www.nhc.gov.cn/ewebeditor/uploadfile/2015/11/20151125105511210.pdf (6 September 2023, date last accessed).

