# Supplementary material for: Malaria cases in China acquired through international travel, 2013–2022
Source: J Travel Med. 2024 Apr 9;31(8):taae056. doi: 10.1093/jtm/taae056 (PMC11646087; doi:10.1093/jtm/taae056)
Supplement: Supplementary_material_taae056 [file supplementary_material_taae056.doc]

**Supplementary material**

Malaria cases in China acquired through international travel, 2013–2022

**Table of Contents**

***S1***. Search strategies from academic databases

***S2***. Links to government reports (Gray literature)

***S3.*** Trend of the imported malaria cases in China from 2013 to 2022

***S4***. Special malaria cases in China from 2013 to 2022

***S5***. Main countries of acquisition of imported malaria cases in China from 2013 to 2022

***S6***. Malaria cases reported by provinces in China from 2013 to 2022

***S1. Search strategies from academic databases***

1. China National Knowledge Infrastructure: <https://chn.oversea.cnki.net/kns/AdvSearch?dbcode=CFLS>

(1) Search strategy: 篇关摘=疟疾 AND 篇关摘=(中国 OR 全国 OR 我国)

(2) Other settings: a) 总库=中文; b) 文献类型=学术期刊; c) 文献分类=医药卫生科技; 4) 来源类别=全部期刊; 5) 时间范围=2013-2022; 6)其他=中英文扩展

2. Wanfang Data

<https://s.wanfangdata.com.cn/advanced-search/paper>

(1) Search strategy: (题名或关键词:(疟疾) AND 题名或关键词:(中国 OR 全国 OR 我国)) OR (摘要:(疟疾) AND 摘要:(中国 OR 全国 OR 我国))

(2) Other settings: a) 文献类型=全部; b)发表时间=2013-2022; 3)智能检索=中英文扩展

3.Chinese Medical Journal Network

<https://www-yiigle-com.libezproxy.must.edu.mo/searchMobile?ind=3>

(1) Search strategy: ((标题=疟疾 AND 标题=(中国 OR 全国 OR 我国)) OR (摘要=疟疾 AND 摘要=(中国 OR 全国 OR 我国))) AND 出版时间=[2013-01-01 TO 2022-12-31]

(2) Other settings: none

4. Google Scholar

<https://scholar.google.com/>

(1) Search strategy: allintitle: malaria China

(2) Other settings: custom range=2013-2022

5. PubMed

<https://pubmed.ncbi.nlm.nih.gov/advanced/>

(1) Search strategy: (((malaria[Title/Abstract]) AND (China[Title/Abstract] OR Chinese[Title/Abstract])) AND (("2013/01/01"[Date - Publication] : "2022/12/31"[Date - Publication]))) AND (English[Language] OR Chinese[Language])

(2) Other settings: none

***S2.*** Links to government reports (Gray literature)

1. China National Health Commission <http://www.nhc.gov.cn/jkj/s5873/201609/3ded209757704ab4871536ca74716661.shtml>

2. China National Disease Control and Prevention Administration: <https://www.ndcpa.gov.cn/jbkzzx/c100016/common/list.html>

3. China CDC

https://www.chinacdc.cn/jkzt/crb/zl/nj/

4. China CDC Data Center

https://www.phsciencedata.cn/Share/ky_sjml.jsp?id=784b16ae-76d4-48a3-9f83-a27591159f59

5. China Customs

<http://wss.customs.gov.cn/wss/yqjc/index.html>

***S3***. Trend of the imported malaria cases in China from 2013 to 2022

| Year | All imported cases | |  | *P.falciparum* | |  | *P.ovale* | |  | *P.vivax* | |  | *P.malariae* | |
| --- | --- | --- | --- | --- | --- | --- | --- | --- | --- | --- | --- | --- | --- | --- |
|  | Trend | *P value* |  | Trend | *P value* |  | Trend | *P value* |  | *Trend* | *P value* |  | *Trend* | *P value* |
| 2013-2019 | ↓ | 0.0442 |  | - | 0.1616 |  | - | 0.0543 |  | ↓ | 0.0182 |  | ↑ | 0.0287 |
| 2013-2022 | ↓ | 0.0054 |  | ↓ | 0.0115 |  | - | 0.5245 |  | ↓ | 0.0034 |  | - | 0.3430 |

[Cuzick's test for trend](https://www.mathworks.com/matlabcentral/fileexchange/22059-cuzick-s-test-for-trend#:~:text=Share%20'Cuzick's%20test%20for%20trend'&text=The%20null%20hypothesis%20of%20no,distribution%20is%20assumed%20for%20errors.)

***S4*.** Special malaria cases in China from 2013 to 2022

There were six cases of transfusion-related malaria, all caused by *P. falciparum*: one case in Jiangsu province in 2016 (the blood donor was infected in Indonesia); two cases in Jiangsu province in 2017 (the blood donor was infected in Equatorial Guinea or Nigeria); one case in Guangdong province in 2017 (the blood donor was infected in South Sudan); and one case each in Shandong and Hunan provinces in 2018 (data on country of acquisition was unavailable). Blood donors were infected with malaria outside China. With the exception of the blood donor for the 2017 Guangdong case, all blood donors were Chinese citizens.

Four introduced *P. vivax* cases were reported without local transmission in Longhui County, Hunan Province in 2018. There were also five cases of long-term dormant *P. malariae* reported in Guangdong province, one case each in 2018 through 2022.

| Year | Transfusion infection | |  | Introduced cases^a^ | |  | Long-term dormant cases | |
| --- | --- | --- | --- | --- | --- | --- | --- | --- |
|  | cases | *Plasmodium* spp. |  | cases | *Plasmodium* spp. |  | cases | *Plasmodium* spp. |
| 2013 | 0 | / |  | 0 | / |  | 0 | / |
| 2014 | 0 | / |  | 0 | / |  | 0 | / |
| 2015 | 0 | / |  | 0 | / |  | 0 | / |
| 2016 | 1 | *P. falciparum* |  | 0 | / |  | 0 | / |
| 2017 | 3 | *P. falciparum* |  | 0 | / |  | 0 | / |
| 2018 | 2 | *P. falciparum* |  | 4 | *P. vivax* |  | 1 | *P. malariae* |
| 2019 | 0 | / |  | 0 | / |  | 1 | *P. malariae* |
| 2020 | 0 | / |  | 0 | / |  | 1 | *P. malariae* |
| 2021 | 0 | / |  | 0 | / |  | 1 | *P. malariae* |
| 2022 | 0 | / |  | 0 | / |  | 1 | *P. malariae* |

*Introduced cases:* First generation of cases acquired via mosquito transmission from imported cases, while the second generation of cases acquired via mosquito transmission from imported cases will be regarded as locally acquired cases.^1^

**Reference**

**S5.** Main countries of acquisition of imported malaria cases in China from 2013 to 2022

| Year | African Countries | | | |  | South-East Asian and other continents | | | |
| --- | --- | --- | --- | --- | --- | --- | --- | --- | --- |
|  | Country | Rank  in the year | Number  of cases | Proportion of imported  cases in the year |  | Country | Rank  in the year | Number  of cases | Proportion of imported  cases in the year |
|  |  |  |  |  |  |  |  |  |  |
| 2013 | Ghana | 1 | 1345 | 33.3% |  | Myanmar | 1 | 608 | 15.0% |
|  | Angola | 2 | 442 | 10.9% |  | Indonesia | 2 | 76 | 1.9% |
|  | Equatorial Guinea | 3 | 296 | 7.3% |  | *n/a* | 3 | *n/a* | *n/a* |
|  | Nigeria | 4 | 217 | 5.4% |  | *n/a* | 4 | *n/a* | *n/a* |
|  | Cameroon | 5 | 100 | 2.5% |  | *n/a* | 5 | *n/a* | *n/a* |
|  | Total | | 2400 | 59.4% |  | Total | | 684 | 16.9% |
|  |  |  |  |  |  |  |  |  |  |
| 2014 | Nigeria | 1 | 330 | 10.9% |  | Myanmar | 1 | 488 | 16.2% |
|  | Equatorial Guinea | 2 | 289 | 9.6% |  | Indonesia | 2 | 142 | 4.7% |
|  | Angola | 3 | 278 | 9.2% |  | *n/a* | 3 | *n/a* | *n/a* |
|  | Ghana | 4 | 188 | 6.2% |  | *n/a* | 4 | *n/a* | *n/a* |
|  | Cameroon | 5 | 170 | 5.6% |  | *n/a* | 5 | *n/a* | *n/a* |
|  | Total | | 1255 | 41.5% |  | Total | | 630 | 20.9% |
|  |  |  |  |  |  |  |  |  |  |
| 2015 | Angola | 1 | 412 | 12.7% |  | Myanmar | 1 | 527 | 16.2% |
|  | Nigeria | 2 | 265 | 8.2% |  | *n/a* | 2 | *n/a* | *n/a* |
|  | Equatorial Guinea | 3 | 248 | 7.6% |  | *n/a* | 3 | *n/a* | *n/a* |
|  | Cameroon | 4 | 242 | 7.5% |  | *n/a* | 4 | *n/a* | *n/a* |
|  | Congo Rep | 5 | 193 | 5.9% |  | n/a | 5 | *n/a* | *n/a* |
|  | Total | | 1360 | 41.9% |  | Total | | 527 | 16.2% |
|  |  |  |  |  |  |  |  |  |  |
| 2016 | Angola | 1 | *n/a* | *n/a* |  | Myanmar | 1 | *n/a* | *n/a* |
|  | Nigeria | 2 | *n/a* | *n/a* |  | *n/a* | 2 | *n/a* | *n/a* |
|  | Cameroon | 3 | *n/a* | *n/a* |  | *n/a* | 3 | *n/a* | *n/a* |
|  | Ghana | 4 | *n/a* | *n/a* |  | *n/a* | 4 | *n/a* | *n/a* |
|  | Guinea | 5 | *n/a* | *n/a* |  | *n/a* | 5 | *n/a* | *n/a* |
|  | Total | | *n/a* | *n/a* |  | Total | | *n/a* | *n/a* |
|  |  |  |  |  |  |  |  |  |  |
| 2017 | Ghana | 1 | 349 | 12.2% |  | Myanmar | 1 | 315 | 11.0% |
|  | Nigeria | 2 | 307 | 10.7% |  | Pakistan | 2 | 67 | 2.3% |
|  | Angola | 3 | 198 | 6.9% |  | Indonesia | 3 | 18 | 0.6% |
|  | Cameroon | 4 | 198 | 6.9% |  | Papua New Guinea | 4 | 22 | 0.8% |
|  | DR Congo | 5 | 153 | 5.4% |  | Guyana | 5 | 6 | 0.2% |
|  | Total | | 1205 | 42.2% |  | Total | | 428 | 15.0% |
|  |  |  |  |  |  |  |  |  |  |
| 2018 | Nigeria | 1 | 350 | 13.1% |  | Myanmar | 1 | 205 | 7.7% |
|  | DR Congo | 2 | 232 | 8.7% |  | Pakistan | 2 | 26 | 1.0% |
|  | Angola | 3 | 204 | 7.6% |  | Cambodia | 3 | 11 | 0.4% |
|  | Ghana | 4 | 166 | 6.2% |  | Papua New Guinea | 4 | 15 | 0.6% |
|  | Cameroon | 5 | 168 | 6.3% |  | *n/a* | 5 | *n/a* | *n/a* |
|  | Total | | 1120 | 41.9% |  | Total | | 257 | 9.6% |
|  |  |  |  |  |  |  |  |  |  |
| 2019 | Nigeria | 1 | 342 | 12.8% |  | Myanmar | 1 | 173 | 6.5% |
|  | DR Congo | 2 | 335 | 12.5% |  | Pakistan | 2 | 21 | 0.8% |
|  | Cote d'Ivoire | 3 | 208 | 7.8% |  | South Korea | 3 | 6 | 0.2% |
|  | Guinea | 4 | 171 | 6.4% |  | Papua New Guinea | 4 | 10 | 0.4% |
|  | Ghana | 5 | 157 | 5.9% |  | *n/a* | 5 | *n/a* | *n/a* |
|  | Total | | 1213 | 45.4% |  | Total | | 210 | 7.9% |
|  |  |  |  |  |  |  |  |  |  |
| 2020 | *n/a* | 1 | *n/a* | *n/a* |  | Myanmar | 1 | 163 | 15.0% |
|  | *n/a* | 2 | *n/a* | *n/a* |  | *n/a* | 2 | *n/a* | *n/a* |
|  | *n/a* | 3 | *n/a* | *n/a* |  | *n/a* | 3 | *n/a* | *n/a* |
|  | *n/a* | 4 | *n/a* | *n/a* |  | *n/a* | 4 | *n/a* | *n/a* |
|  | *n/a* | 5 | *n/a* | *n/a* |  | *n/a* | 5 | *n/a* | *n/a* |
|  | Total | | *n/a* | *n/a* |  | Total | | 163 | 15.0% |
|  |  |  |  |  |  |  |  |  |  |
| 2021 | DR Congo | 1 | *n/a* | *n/a* |  | Myanmar | 1 | 127 | 15.9% |
|  | Guinea | 2 | *n/a* | *n/a* |  | *n/a* | 2 | *n/a* | *n/a* |
|  | Nigeria | 3 | *n/a* | *n/a* |  | *n/a* | 3 | *n/a* | *n/a* |
|  | Cameroon | 4 | *n/a* | *n/a* |  | *n/a* | 4 | *n/a* | *n/a* |
|  | Congo Rep | 5 | *n/a* | *n/a* |  | *n/a* | 5 | *n/a* | *n/a* |
|  | Total | | *n/a* | *n/a* |  | Total | | 127 | 15.9 |
|  |  |  |  |  |  |  |  |  |  |
| 2022 | Guinea | 1 | *n/a* | *n/a* |  | Myanmar | 1 | *n/a* | *n/a* |
|  | Nigeria | 2 | *n/a* | *n/a* |  | *n/a* | 2 | *n/a* | *n/a* |
|  | Congo Rep | 3 | *n/a* | *n/a* |  | *n/a* | 3 | *n/a* | *n/a* |
|  | Cote d'Ivoire | 4 | *n/a* | *n/a* |  | *n/a* | 4 | *n/a* | *n/a* |
|  | *n/a* | 5 | *n/a* | *n/a* |  | *n/a* | 5 | *n/a* | *n/a* |
|  | Total | | *n/a* | *n/a* |  | Total | | *n/a* | *n/a* |
|  |  |  |  |  |  |  |  |  |  |

***S6*.** Malaria cases reported by provinces in China from 2013 to 2022

| Year | Province | Rank in the year | Number of cases | Proportion of all cases  in the year |
| --- | --- | --- | --- | --- |
|  |  |  |  |  |
| 2013 | Guangxi | 1 | 1251 | 30.3% |
|  | Yunnan | 2 | 576 | 14.0% |
|  | Jiangsu | 3 | 341 | 8.3% |
|  | Sichuan | 4 | 238 | 5.8% |
|  | Zhejiang | 5 | 208 | 5.0% |
|  | Total | | 2614 | 63.4% |
|  |  |  |  |  |
| 2014 | Yunnan | 1 | 533 | 17.3% |
|  | Jiangsu | 2 | 355 | 11.5% |
|  | Sichuan | 3 | 266 | 8.6% |
|  | Henan | 4 | 216 | 7.0% |
|  | Zhejiang | 5 | 215 | 7.0% |
|  | Total | | 1585 | 51.4% |
|  |  |  |  |  |
| 2015 | Yunnan | 1 | 606 | 18.4% |
|  | Jiangsu | 2 | 405 | 12.3% |
|  | Sichuan | 3 | 290 | 8.8% |
|  | Guangxi | 4 | 236 | 7.2% |
|  | Shandong | 5 | 212 | 6.4% |
|  | Total | | 1749 | 53.1% |
|  |  |  |  |  |
| 2016 | Yunnan | 1 | 413 | 12.4% |
|  | Sichuan | 2 | 327 | 9.8% |
|  | Jiangsu | 3 | 308 | 9.3% |
|  | Guangxi | 4 | 305 | 9.2% |
|  | Shandong | 5 | 256 | 7.7% |
|  | Total | | 1609 | 48.4% |
|  |  |  |  |  |
| 2017 | Guangxi | 1 | 382 | 13.4% |
|  | Yunnan | 2 | 325 | 11.4% |
|  | Jiangsu | 3 | 239 | 8.4% |
|  | Shandong | 4 | 209 | 7.3% |
|  | Sichuan | 5 | 209 | 7.3% |
|  | Total | | 1364 | 47.8% |
|  |  |  |  |  |
| 2018 | Guangxi | 1 | 254 | 9.5% |
|  | Jiangsu | 2 | 243 | 9.1% |
|  | Shandong | 3 | 233 | 8.7% |
|  | Sichuan | 4 | 221 | 8.3% |
|  | Yunnan | 5 | 213 | 8.0% |
|  | Total | | 1164 | 43.6% |
|  |  |  |  |  |
| 2019 | Jiangsu | 1 | 244 | 9.1% |
|  | Shandong | 2 | 228 | 8.5% |
|  | Henan | 3 | 227 | 8.5% |
|  | Guangdong | 4 | 206 | 7.7% |
|  | Sichuan | 5 | 199 | 7.4% |
|  | Total | | 1104 | 41.2% |
|  |  |  |  |  |
| 2020 | Guangdong | 1 | *n/a* | *n/a* |
|  | Yunnan | 2 | *n/a* | *n/a* |
|  | Jiangsu | 3 | *n/a* | *n/a* |
|  | Sichuan | 4 | *n/a* | *n/a* |
|  | Shandong | 5 | *n/a* | *n/a* |
|  | Total | | 570 | 52.5% |
|  |  |  |  |  |
| 2021 | Guangdong | 1 | *n/a* | *n/a* |
|  | Yunnan | 2 | *n/a* | *n/a* |
|  | Shanghai | 3 | *n/a* | *n/a* |
|  | Sichuan | 4 | *n/a* | *n/a* |
|  | Zhejiang | 5 | *n/a* | *n/a* |
|  | Total | | 480 | 60.1% |
|  |  |  |  |  |
| 2022 | Guangdong | 1 | 182 | 21.5% |
|  | Yunnan | 2 | 136 | 16.1% |
|  | Sichuan | 3 | 72 | 8.5% |
|  | Zhejiang | 4 | 64 | 7.6% |
|  | Henan | 5 | 59 | 7.0% |
|  | Total | | 513 | 60.7% |
|  |  |  |  |  |
